# X-ray beam-shaping *via* deformable mirrors: surface profile and point spread function computation for Gaussian beams using physical optics

**DOI:** 10.1107/S1600577517014035

**Published:** 2018-01-01

**Authors:** D. Spiga

**Affiliations:** a INAF – Osservatorio Astronomico di Brera, Via Bianchi 46, Merate, Italy

**Keywords:** X-ray mirrors, active optics, beam-shaping, physical optics

## Abstract

X-ray mirrors with high focusing performances are commonly used in different sectors of science, such as X-ray astronomy, medical imaging and synchrotron/free-electron laser beamlines. While deformations of the mirror profile may cause degradation of the focus sharpness, a deliberate deformation of the mirror can be made to endow the focus with a desired size and distribution, *via* piezo actuators. The resulting profile can be characterized with suitable metrology tools and correlated with the expected optical quality *via* a wavefront propagation code or, sometimes, predicted using geometric optics. In the latter case and for the special class of profile deformations with monotonically increasing derivative, *i.e.* concave upwards, the point spread function (PSF) can even be predicted analytically. Moreover, under these assumptions, the relation can also be reversed: from the desired PSF the required profile deformation can be computed analytically, avoiding the use of trial-and-error search codes. However, the computation has been so far limited to geometric optics, which entailed some limitations: for example, mirror diffraction effects and the size of the coherent X-ray source were not considered. In this paper, the beam-shaping formalism in the framework of physical optics is reviewed, in the limit of small light wavelengths and in the case of Gaussian intensity wavefronts. Some examples of shaped profiles are also shown, aiming at turning a Gaussian intensity distribution into a top-hat one, and checks of the shaping performances computing the at-wavelength PSF by means of the *WISE* code are made.

## Abstract 2

A method to obtain the deformation profile to be imparted to an X-ray mirror in order to turn a Gaussian intensity distribution into any assigned point spread function, and how to easily check the result using physical optics.

## 1. Introduction   

A great effort has been deployed in recent years to manufacture X-ray mirrors with high resolving power, both in the domain of X-ray astronomy and on-ground facilities such as synchrotrons and free-electron lasers (FELs). The resolving power is the size of the focal spot, usually expressed in terms of point spread function (PSF). The shape of the PSF is the combination of the intrinsic aperture diffraction and of the mirror’s fabrication defects, which in turn include geometric deformations and surface finishing imperfections.

The angular resolution in astronomical X-ray telescopes (VanSpeybroeck & Chase, 1972 ▸), *i.e.* the ability to separate individual sources in crowded fields, currently ranges from 0.5 arcsec for the Chandra X-ray Observatory (Weisskopf, 2012 ▸) to 16 arcsec for eROSITA (Burwitz *et al.*, 2013 ▸; to be launched in 2018). The different value stems from the different manufacturing technique, the direct figuring/polishing of thick mirrors for Chandra and the nickel electroforming of thin mirror shells for eROSITA. An optical system design based on thin mirror shells is, in particular, suitable for obtaining large collection areas because it enables a dense nesting of a number of shells, which makes it possible to detect distant and faint X-ray sources. Nonetheless, thin mirror shells are more subject to deformations, and this is the reason why a higher nesting density is always obtained at the expense of the focusing accuracy, which remains – as of today – limited by profile and surface defects.

Optical systems for terrestrial X-ray sources typically focus, collimate and deflect intense X-ray beams; therefore, these mirrors do not require a large effective area. Nesting is not required either, so the mirrors in the optical setup can be thick in order to maximize their mechanical stiffness. Hence, efforts can be made aiming at the best possible surface finishing and to achieve a mirror profile as close as possible to the nominal one: *e.g.* an ellipsoid or a K–B system (Wolter, 1952 ▸; Kirkpatrick & Baez, 1948 ▸). The achieved profile accuracy can be so high that the PSF becomes limited by the deformation induced by the supporting system or the temperature instability, and therefore the mirror shape has to be actively corrected at the operation time. This can be done by equipping the mirror with a system of benders (Raimondi *et al.*, 2014 ▸) for overall curvature correction or bimorph actuators to achieve the correction over shorter spatial scales (Signorato *et al.*, 1998 ▸). In the best performing cases, with a focal spot of a few nanometers, these optics are nowadays approaching the diffraction limit in soft X-rays (Idir *et al.*, 2010 ▸). In practice, there are practical limitations to the corrections that can be operated *via* actuators, such as the maximum strain they can exert on the mirrors, the difficulty modeling the surface at junctions between the actuators, and the realistic determination of the voltages to be applied (Vannoni *et al.*, 2015 ▸).

However, for some applications the focused beam’s maximum sharpness is not required. Rather, a deliberate deformation can be imparted to the mirror longitudinal profile in order to re-distribute the power intercepted by the mirror and endow the PSF with a given profile. Beam-shaping capabilities are possessed, for example, at the EIS-TIMEX beamline of FERMI (Svetina *et al.*, 2012 ▸), where an initially Gaussian intensity distribution is turned into a top-hat PSF on the focal plane. The mirror shape was, however, found by a trial-and-error approach and the search will need to be iterated every time the required PSF profile is changed. In contrast, an analytical tool able to return the mirror bending for any required PSF would be much more effective and easy to implement.

**Figure 1:**
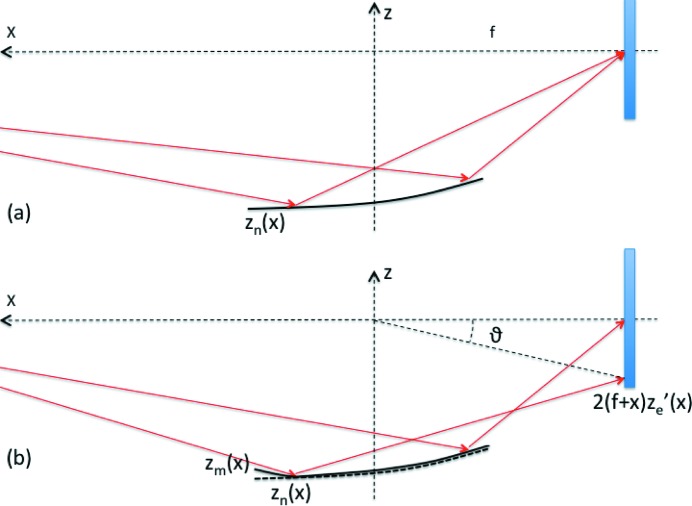
(*a*) The nominal longitudinal mirror profile 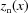 focuses exactly the incident beam to a focal point. (*b*) A deliberate deformation 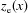 is superposed to 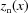 in order to change the intensity distribution on the focal plane. The longitudinal profile is now 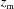 = 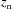 + 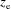.

While the problem of computing the PSF generated by a perturbed mirror profile is widely studied, *e.g.* using dedicated ray-tracing routines, the inverse problem is much more delicate to treat. This point is illustrated in Fig. 1 ▸: if 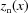 is the mirror profile that exactly focuses the incident beam (*e.g.* an ellipse for terrestrial sources, a parabola for an infinitely distant source), we can alter this profile adding a small perturbation 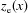 aiming at changing the intensity distribution throughout the PSF. If geometric optics can be used, the PSF can be computed from the perturbed profile 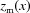 = 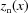 + 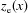, and it is only a function of the incidence angles distribution along *x*. However, if the slopes are small enough, the incidence angle variation can be approximated with 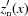 + 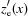. Since 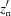 returns a perfect focus by hypothesis, the PSF should be a function of the sole 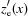, *i.e.* of the ‘profile error’ 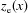. A similar argument based on physical optics can be used to reach the same conclusion (Raimondi & Spiga, 2015 ▸). Clearly, there is no unique mirror 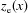 that returns an assigned PSF; rather, infinite possible profile errors can be associated with a specified PSF form.

Fortunately, for the present scope of beam-shaping, we only need to derive *a single* 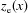 function to yield the selected PSF. In particular, we can select the mirror profile with the simplest properties, *e.g.* without concavity changes. Based on this hypothesis and assuming the geometric optics to be completely applicable, we have elaborated a one-dimensional formalism (Spiga *et al.*, 2013*a* ▸) to compute a profile deformation from any desired PSF and an arbitrary intensity distribution incident on the mirror. Clearly, this approach always returns *continuous* deformations and might not always be compatible with the bending/bimorph system available for the optical system in use, typically consisting of *discrete* elements. Other authors (Laundy *et al.*, 2015 ▸; Sutter *et al.*, 2016 ▸) have elaborated methods to account for the finite extent of actuators and reproduce a top-hat PSF as much as possible. However, in the remainder of this paper we do not consider this limitation, but approach the solution to the beam-shaping problem assuming a technology enabling the application of a continuous strain distribution along the mirror profile to become available soon. In practice, the present treatment applies to a very large array of very small actuators such as the one realised by Reid *et al.* (2014 ▸).

Our computation based on geometric optics did not account for the spatial coherence of the incoming wavefront, therefore neglecting the aperture diffraction effects, and also those of the source dimension. In this paper, we re-derive our previous results in the framework of the more general treatment of physical optics (§2), in the frequent case of small λ values and for Gaussian intensity wavefronts, typical of FELs in the fundamental propagation mode (Raimondi *et al.*, 2013 ▸). This not only makes us understand to which approximation the beam-shaping formulae are valid but also offers the opportunity to check the real beam-shaping performances accounting for the coherence of the incident wavefront, which automatically contains all the information regarding the source profile. To this end, we make use of the one-dimensional Fresnel diffraction formalism (Spiga & Raimondi, 2014 ▸; Raimondi & Spiga, 2015 ▸) implemented in the *WISE* code (*Wavefront propagatIon Simulation codE*). This is described in §3 along with some computation examples.

## 2. PSF shaping with deformable mirrors under Gaussian illumination   

### 2.1. General PSF expression in far-field conditions   

As stated in §1, we hereby assume the nominal mirror shape 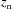 to exactly focus the source into the origin of the reference frame, and the perturbation imparted to the mirror profile still be described by 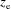 (Fig. 1 ▸). If the wavefront has uniform amplitude, the PSF at the light wavelength λ has the expression (Raimondi & Spiga, 2015 ▸) in the far-field approximation,
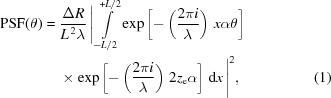
where α is the grazing incidence angle, λ is the light wavelength, θ is the angular distance from the center of the ideal focal spot, and 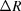 ≃ 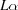 is the mirror aperture in the plane normal to the beam direction. We assume *L*, the mirror length, to be much smaller than the distance to the observation plane. With respect to the expression in the original paper, we have changed the notation replacing 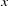 by 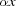, because *x* now denotes the coordinate over the mirror length rather than the entrance pupil.

If the wavefront is *nonuniform*, *i.e.* characterized by a variation in intensity with *x*, the PSF expression is easily adapted,
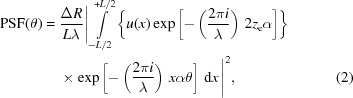
where 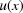 is a real function representing the electric field amplitude at the mirror surface, and is supposed to be normalized to 1 in intensity,
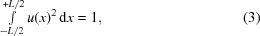
having absorbed a constant mirror reflectivity into the 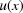 definition. The units of 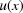 are those of 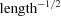, and for this reason *L* appears at the first power in equation (2). The expression {…} in equation (2) is known as the complex pupil function (CPF).

We now extend the integration range to 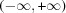, setting the 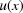 value to zero outside the mirror length. Then we apply the Wiener–Khintchine theorem to equation (2), and remove the squared module,
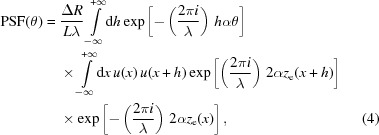
where the second integral is the autocorrelation function of the CPF and *h* is the ‘lag’ between the amplitude profile 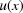 and its shifted copy 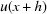. Re-arranging the terms, we can rewrite equation (4) in the form
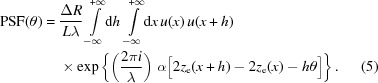
We are not including in 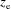 any roughness (*i.e.* fractal) component, therefore the profile has a derivative everywhere and we are allowed to write 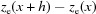 = 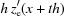, with 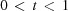. In this way, we obtain
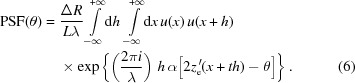



### 2.2. The case of Gaussian beams at small λ   

We now limit ourselves to the case of a Gaussian anisotropic beam, *i.e.*

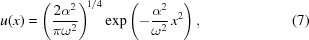
where the normalization factor was chosen for 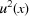 to fulfill equation (3), and 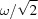 is the amplitude width r.m.s. at the mirror location, measured in the plane transversal to the propagation direction. The Gaussian beam propagation theory shows that 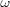 ≃ 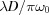, where 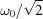 is the source width r.m.s. and *D* is the mirror–source distance (Raimondi *et al.*, 2013 ▸). Substituting the expression of 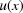 into equation (6) and exchanging the integration order, we obtain
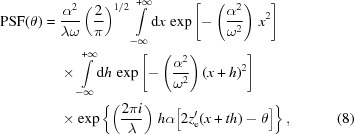
where we have substituted 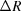 = 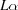. We additionally assume that for X-rays the wavelength is much smaller than the coherence length of the surface, *i.e.* 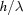 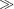 1, and therefore only a small interval of 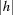 ≃ 0 contributes to the integral. Hence, we can write 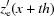 ≃ 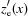. If we now set 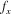 = 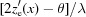, equation (8) reads
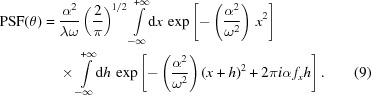
Changing the integration variable 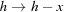 in the second integral, after some handling we remain with
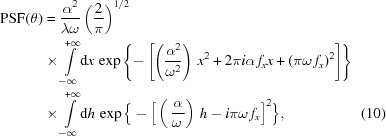
and the integral in 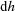 clearly equals 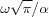, so we obtain
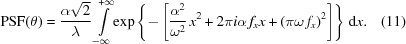
In the case of a perfect mirror, 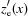 = 0, 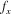 = 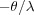 and equation (11) yields
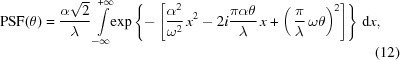
which returns, after some easy passages,
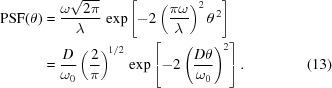
As expected, the coherent propagation of a Gaussian wavefront returned an image that reproduces the profile of the source, *i.e.* with the same characteristic width 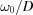 = 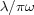. In general, the integral in equation (11) cannot be solved analytically because 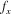 depends on the functional form of 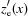.

### 2.3. Beam-shaping formulae in the geometric optic limit   

By some manipulation, equation (11) can be put in the form
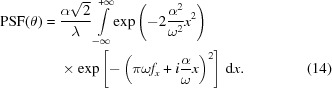
At sufficiently high energies we can assume 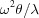 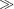 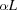, so we can neglect the term 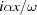 in the second exponent. We remain with
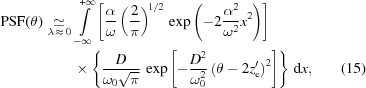
where we have made use of the relation 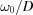 = 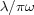. The second factor in the integral is simply the amplitude profile of the source seen to a distance *D* and ‘distorted’ by the profile perturbation. Equation (15) does no longer depend on λ, therefore the approximation we made is the passage to the geometric optics. We also note that in the limit of a point source 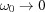, the source profile becomes a Dirac delta function, and equation (15) assumes the simpler form
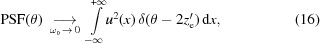
which simply represents the re-distribution of the wavefront intensity according to the function 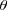 = 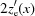.

We now make use of the additional hypothesis, *that* 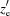 *be an increasing function of *x**. This means that the function 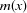 = 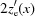 can be inverted, so we can change the integration variable to *m* in equation (15),
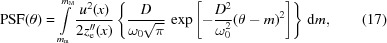
where *m* varies between the minimum 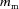 = 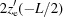 and the maximum value 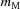 = 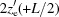.

Finally, in the limit of a point-like source, the Gaussian term in equation (17) becomes a delta and the result becomes
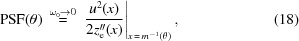
for all values of θ that correspond to some *x* *via* the equation
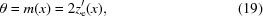
which sets a one-to-one mapping between the location on the mirror profile, *x*, and the angular coordinate of the PSF, θ. This is exactly the PSF expression we already derived (Spiga *et al.*, 2013*a* ▸) *via* a purely geometric reasoning. This approach, based on curvature detection, has been also extended to the intra-focal configuration for mirror shape reconstruction under X-ray illumination (Spiga *et al.*, 2013*b* ▸).

The monotonic trend of 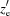 clearly implies that 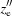 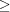 0 over all the entire mirror length. At the locations where 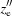 = 0, the PSF exhibits either cusps or Dirac delta-like peaks, depending on whether 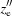 vanishes either at isolated points or in mirror segments [see Spiga *et al.* (2013*a* ▸) for further details]. In any case, the PSF provided by equation (18) is normalized,
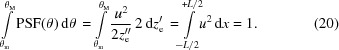
Using equation (19), we can rewrite equation (18) as follows,
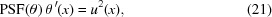
and the solution to this equation is
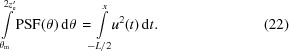
The validity of equations (18) and (22) is not limited to the case of Gaussian wavefronts. We can now replace any two functional forms for PSF(θ) and 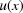 – non-negative and fulfilling the normalization conditions (3) and (20) – into equation (22) and solve it for 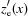. Doing this, problems may arise whenever 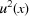 = 0 in one or more intervals of the mirror profile, or should the PSF be zero in some region of the focal plane (as in the example shown in Fig. 6). Fortunately, these special cases can be managed easily: a detailed discussion on these topics is reported by Spiga *et al.* (2013*a* ▸).

We finally notice that the treatment exposed here is valid in the limit that the distance to the observation plane is much larger than *L*. Should this condition be violated, we can account for the finite focal length correcting equations (18) through (22) with a proper asymmetry factor. Once again, a detailed discussion on this topic can be retrieved from Spiga *et al.* (2013*a* ▸).

## 3. Some computation examples   

**Figure 2:**
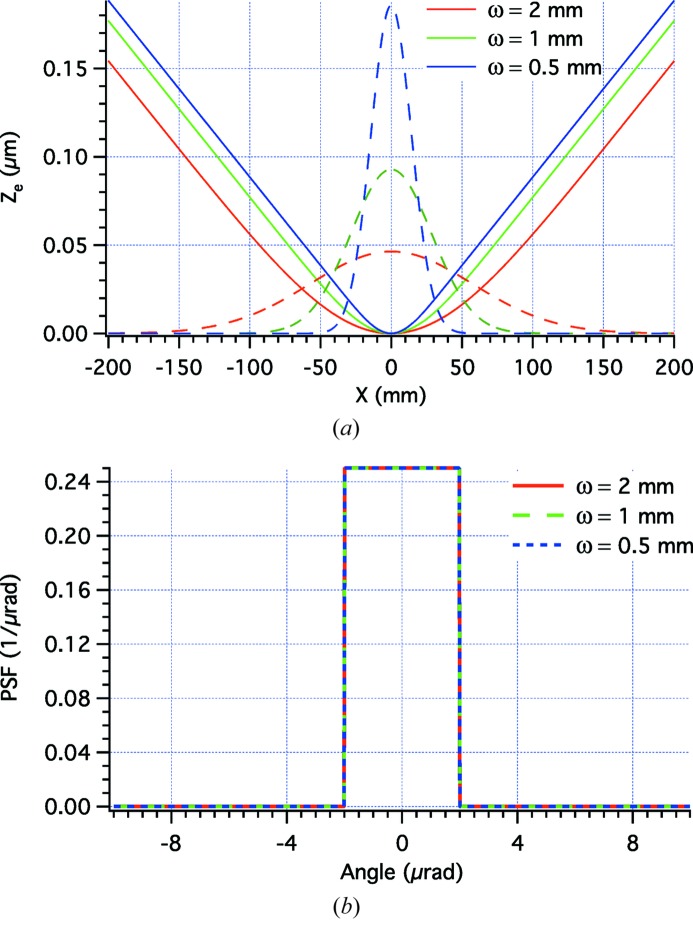
(*a*) Solid lines: the profile deformations required to turn Gaussian amplitude distributions (dashed lines) of variable ω [equation (7)] into the same top-hat PSF, shown in (*b*). We have adopted the values *L* = 400 mm, α = 1° and equation (23) with *w* = 4 µrad; the Gaussian amplitude graphs have arbitrary units.

We now consider some examples of application of the beam-shaping formulae reported in the previous section. We initially consider the most frequently requested PSF shape, *i.e.* the top-hat one,
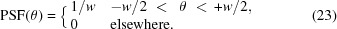
Replacing now the expressions of 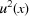 [equation (7)] and of the top-hat PSF into equation (22) and solving numerically for various ω values, we obtain different 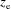 profiles as shown in Fig. 2(*a*) ▸, always aiming at the same top-hat PSF (Fig. 2*b* ▸).

**Figure 3:**
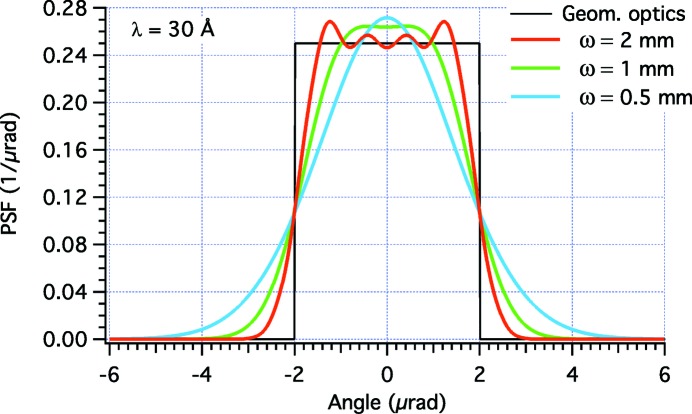
*WISE* simulation of PSFs for the profile perturbations shown in Fig. 2 ▸ at λ = 30 Å, at variable ω values. The characteristic width of the Gaussian source 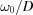 equals 0.48, 0.96 and 1.92 µrad in the three respective cases.

**Figure 4:**
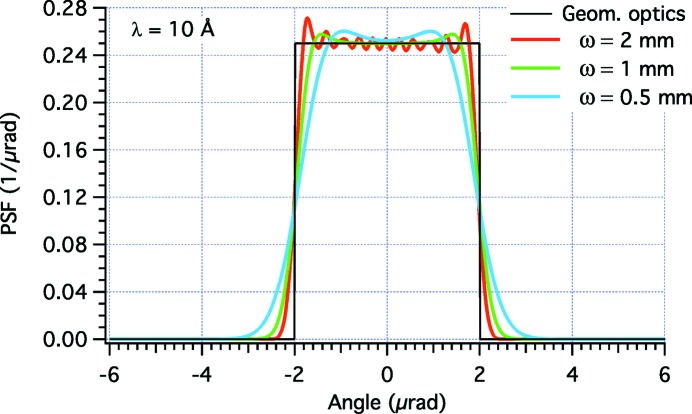
*WISE* simulation of PSFs for the profile perturbations shown in Fig. 2 ▸ at λ = 10 Å, at variable ω values. The characteristic width of the Gaussian source 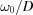 equals 0.16, 0.32 and 0.64 µrad in the three respective cases.

**Figure 5:**
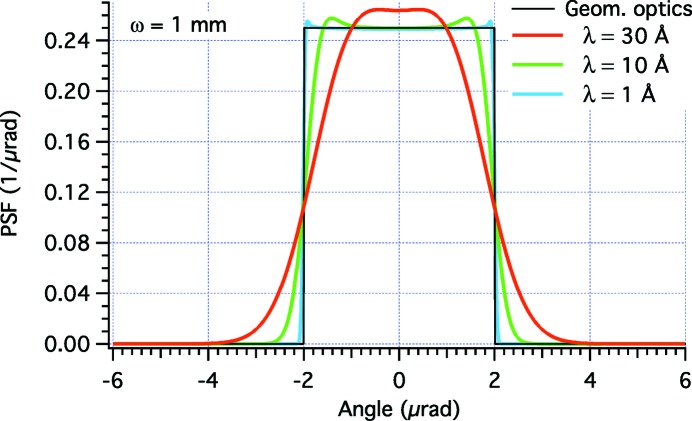
*WISE* simulation of PSFs for the profile perturbation shown in Fig. 2 ▸ in the case ω = 1 mm, at decreasing λ values. The characteristic width of the Gaussian source 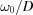 equals 0.96, 0.32 and 0.03 µrad for the three wavelengths, respectively.

We may now want to check the effectiveness of the shaped profiles using equation (1) implemented in the *WISE* code (Raimondi & Spiga, 2015 ▸). In these simulations, we have superimposed the perturbations displayed in Fig. 2(*a*) ▸ to an elliptical mirror with a sagittal curvature radius 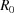 = 1165 mm, a distance *f* = 40 m to the focal plane, and a distance *D* = 200 m to the light source. The incidence angle is α = 1°. The PSFs are computed at λ = 30 Å in Fig. 3 ▸ and at λ = 10 Å in Fig. 4 ▸. We immediately note that the physical optics simulations deviate from the targeted PSF because the edges are not abrupt, and because there are diffraction fringes of variable amplitude and frequency. The best approximation to the top-hat profile is clearly obtained at higher energy with the broader beam (the case ω = 2 mm in Fig. 4 ▸). This corresponds to our findings of §2.3 that the geometric optics results are approached in the limits of small λ and 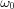. The smallest 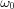 values, corresponding to the larger apertures of the beam on the mirror, cause the lower departures from the geometric optics because the beam is diffracted through a broader ‘slit’ (roughly 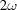 wide). The contrary occurs at large values of λ and 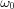, corresponding to a diffraction through a small 2ω. This is seen for example in Fig. 5 ▸, where we displayed the PSF evolution for a hypothetically fixed value ω = 1 mm and variable X-ray wavelength: in the case of λ = 30 Å, the top-hat profile is completely hidden by the Gaussian profile of the source. Therefore, using the physical optics method for PSF computation we do not even need to convolve the PSF with the demagnified source profile because all of the information regarding the source size is already included in the width of the Gaussian wavefront at the mirror. This is possible because the wavefront is assumed to be highly spatially coherent, as typical of FELs and of most beamlines in synchrotron facilities.

**Figure 6:**
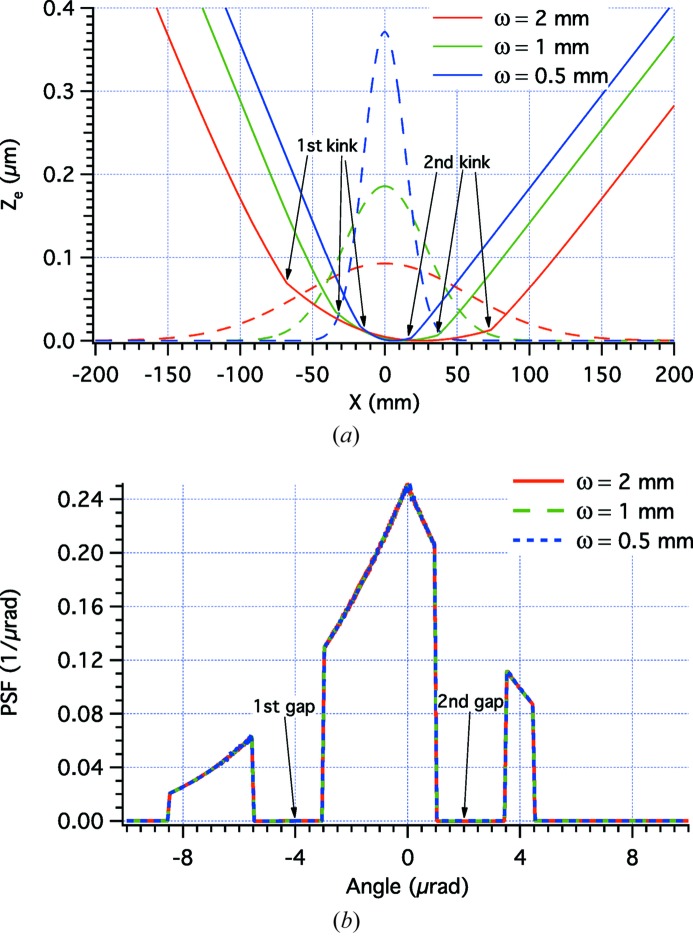
(*a*) Solid lines: the profile deformations required to turn Gaussian amplitude distributions (dashed lines) of variable ω [equation (7)] into the same PSF (*b*), consisting of three separated blocks. As we did in the top-hat PSF simulations, we used the values *L* = 400 mm, α = 1° and the Gaussian amplitude graphs have arbitrary units. The full range of the profiles is not shown in order to draw attention to the shaped center, where the beam intensity is relevant: the omitted parts correspond to the tails of the Gaussian and simply consist of straight (non-shaping) segments. The arrows mark the profile kinks needed to make the gaps in the PSF.

We have hitherto considered the case of a top-hat PSF, but the method exposed in this paper equally works for *any* non-negative and normalized PSF. For example, the PSF shown in Fig. 6(*b*) ▸, made of three separated peaks, can be easily reproduced – from Gaussian wavefronts at the mirror – by imparting deformations 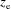 as shown in Fig. 6(*a*) ▸: also in this case, the profile shape was adapted to the actual width of the Gaussian beam in order to always return the same PSF. In this case, the solution of equation (22) may pose some problems because the left-hand side becomes constant in correspondence to the PSF gaps, and therefore infinite values of 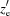 are possible. This issue can be managed adopting for 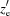 the limit value at the closest edge of the intervals where the left-hand of equation (22) is constant. This usually results in 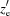 discontinuities, and consequently kinks in 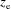 just like the ones visible in Fig. 6(*a*) ▸.

**Figure 7:**
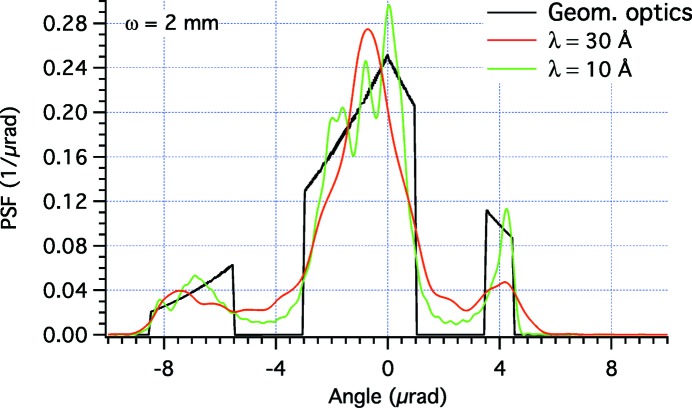
*WISE* simulation of PSFs for the profile perturbation shown in Fig. 6(*a*) ▸ in the case ω = 2 mm, at two different λ values. The characteristic width of the Gaussian source 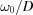 equals 0.48 and 0.16 µrad in the two respective cases. Simulations at two smaller values of λ have been included in Fig. 8 ▸ to avoid confusion in the figure.

**Figure 8:**
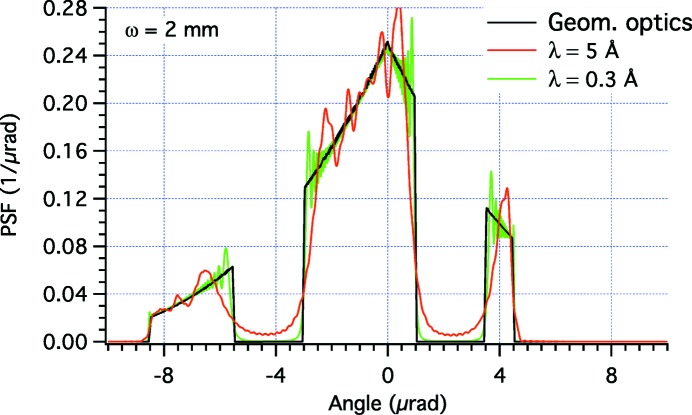
*WISE* simulation of PSFs for the profile perturbation shown in Fig. 6(*a*) ▸ in the case ω = 2 mm, at two λ values smaller than the ones used in Fig. 7 ▸. The characteristic width of the Gaussian source 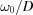 equals 0.08 and 0.005 µrad in the two respective cases.

Also with this profile deformation, we have verified the real PSF using the *WISE* code at different X-ray wavelengths (Figs. 7 ▸ and 8 ▸), limited to the case in which we expect to minimize the diffractive effects, *i.e.* ω = 2 mm. In reality, the diffraction features are even more apparent in this example than in the top-hat profile considered beforehand, because the PSF is more peaked toward the center. In the simulation (Fig. 7 ▸) at 30 Å, the three peaks appear confused in one because the angular diameter of the source is of the order of 1 µrad, close to the dimension of the gaps (2 µrad) expected geometrically. At 10 Å, the structures of the individual peaks have started emerging (Fig. 7 ▸), but are still partially hidden by prominent diffraction fringes. At higher energies (Fig. 8 ▸), there are still fringes but the overall PSF is now following better the expected PSF profile, and the gaps between the three blocks are now better visible. Finally, at λ = 0.3 Å, the geometric pattern is well reproduced in the simulation (Fig. 8 ▸), even if some edge effects can still be seen. We can therefore state that, for a PSF of this kind and a Gaussian beam of this width, the performances of the deterministic beam-shaping method are satisfactory at energies beyond 40 keV.

## 4. Conclusions   

In this paper, we have provided a complete derivation of the analytical formulae [equations (18) and (22)] for the deterministic beam-shaping of coherent Gaussian wavefronts, starting from the physical optics expressions of the PSF, in the limit of small wavelengths and small X-ray sources. The method is limited to continuous profile deformations and does not account for the discreteness of the actuators that, in the current piezo technology, can be used to actively correct the shape of deformable mirrors. However, the analytical method allows us to avoid complex search algorithms and returns, at sufficiently high X-ray energies, a PSF very close to the required one, as we could easily verify by means of the *WISE* code. Therefore, this approach can effectively provide an initial profile correction to be later refined by numerical algorithms in order to account for the mentioned limitations (finite dimensions of the source and of the piezoelectric elements). Future work will be aimed at the search of more general formulae to describe the beam-shaping formalism at the wavelength in use, without the need to pass to the geometric optics limit.
